# Development of self-critical abilities and values in students using digital games-based learning

**DOI:** 10.3389/fpsyg.2023.1193244

**Published:** 2023-10-19

**Authors:** Chunning Zuo

**Affiliations:** School of Marxism Studies, Shaanxi Technical College of Finance and Economics, Xianyang, Shaanxi, China

**Keywords:** digital games, innovative technologies, motivation, self-criticism, youth psychology

## Abstract

Young people are the driving force of society. Therefore, the well-being of society itself depends on what values and ideals they carry into adulthood. The purpose of this study was to identify and describe the values of contemporary Chinese youth in the context of their future life goals and to analyze how self-criticism shapes students’ depressive tendencies with the use of digital games. This study was conducted at Shaanxi Technical College of Finance and Economics with 157 Chinese students between the ages of 18 and 25. One hundred nine girls and forty-eight boys were randomly selected for the study. The study was conducted in a Chinese cultural context using the method of experiment with a survey, statistical, and correlational data analysis. Correlation analysis showed that the correlation between self-criticism and N/NE is strong (rs ¼ 0.50–0.65), but it was most related to the positive emotional component of E/PE. Thus, no obvious and serious reasons for the development of depression in young people were found. The results showed that digital games cannot influence the development of self-critical abilities of students, however, they can be a high-quality tool in psychological work with students to determine depressive moods, an overestimated level of self-criticism, and other problems that prevent them from learning. This article has implications for further research in education, as it may provide a basis for developing and improving new methods of constructing curricula. They can be aimed at defining special attention to the psychological state of students to prevent depressive states caused by high levels of self-criticism.

## 1. Introduction

Values and beliefs serve many functions for both individuals and society as a whole. They play a crucial role in determining personal goals, plans, and behavior, as well as affecting health and well-being (Schoon and Mortimer, 2017). Young people need to be morally driven to develop first themselves and then society. Without this, leadership becomes futile because it is inextricably linked to achieving goals, and those goals can be achieved by all members of society. The development of society depends on the values that people bring to it. Prosperity means success and new achievements, and in order for any society to have these qualities, young people must learn the right values (Gibson and Trnka, 2020). This will help them become positively oriented leaders of today. Societal prosperity refers to the overall well-being, success, and prosperity of its members. It goes beyond just economic wealth and material goods to encompass the various aspects of life that contribute to the happiness and quality of life of people in society (Joshanloo et al., 2019). Values serve as the foundation for prosperity and success in society. When people hold positive values such as honesty, empathy, responsibility, and respect, they contribute to a harmonious and productive social environment. These values are essential for building trust, cooperation, and a sense of collective responsibility (Gunawan and Gunawan, 2019). It follows that young people are the engine of society. Teenagers are usually very energetic and are always willing to do more than it takes to achieve what they believe in and adhere to. The Oxford Dictionary for Advanced Learners defines youth as “the time of life when a person is young, especially the time before a child becomes an adult” (Colman, 2015).

Understanding how young people work toward their goals in life can provide guidance for developing a positive appreciation of their values. Multiple dimensions of human development in the context of age distribution must be considered (Wang et al., 2022). Among all global life transitions, the transition to adulthood ranks very high in importance, complexity, and uniqueness. It involves a series of processes involving the assumption of new social roles in education, work, life, relationships, etc. (Bronk et al., 2019). The transition to adulthood is a critical stage and turning point in young people’s lives, when external shocks directly affect their potential and formation (Fletcher, 2015). While in childhood and adolescence individual lives are organized by institutional structures (compulsory schooling and legal frameworks to support and protect children), most of these structures fall away when children reach adulthood (Schoon and Mortimer, 2017).

The potential of digital technologies as a co-facilitator in the implementation of learning is a key factor in facilitating contextual learning (Bayne, 2015). They intertwine in political, economic, cultural, and material contexts. The number of studies on the effectiveness of digital games in teaching critical thinking is increasing (Haßler et al., 2016; Castañeda and Selwyn, 2018). This is because digital game-based learning offers students an environment to engage in critical thinking. Students use analytical skills and apply critical thinking to the game as they navigate their game experience. For example, they must rely on critical thinking skills such as analysis and evaluation to assess the situation and weigh the decisions they intend to make. The link between students’ self-critical abilities and values and digital game-based learning lies in the potential of this educational approach to promote personal growth, self-awareness, and the development of positive values. Understanding how game-based digital learning can impact students’ self-critical abilities and values can help educators design more effective and engaging learning experiences. By harnessing the potential of educational games, educators can create a positive and supportive environment for holistic development.

### 1.1. Literature review

#### 1.1.1. Level of self-critical abilities of youth

The United Nations General Assembly defines youth in the age group of 18 to 34. The Commonwealth Youth Program sets the age group at 15 to 29, while the Danish Youth Council places young people in the age group of 15 to 34 (Harada, 2023). Judging by the above age groups, one cannot specify a particular age for a person to be identified as a youth. It would be identified as a group of young people who exhibit the following behavioral characteristics: a strong desire to move up the ladder; a tendency toward idealism as a result of established values conveyed to them at an earlier age by role models (Gabrielli et al., 2022). The desire to live according to this pattern can cause frequent frustration and anxiety, as idealism is opposed to the cold realism of everyday existence. Character is interpreted by psychologists as consisting of positive and socially significant elements of personality (Roberts and Nickel, 2017). This social value implies character as elements of personality with a moral component. That is, personality tends to develop in socially desirable directions with greater social awareness and contribution (Hilliard et al., 2014).

In an information-rich, knowledge-driven world, critical thinking has become a critical civic literacy factor for students to adapt to society (Bespalov et al., 2017). The more meaningful knowledge that can be taught in the classroom, the greater the critical thinking need for students to absorb and retain that knowledge (Brown et al., 2020). Thus, modern education has gradually shifted its focus to the development of critical thinking among students (Chen and Wu, 2023). Differentiation of critical thinking depending on the level of education is also attracting more attention in the academic community. Young people’s interaction with negative events, along with high levels of self-criticism, can exacerbate depressive symptoms. Levels of self-criticism may appear to interact with negative interpersonal situations for the same reason (Auerbach, 2015). Self-criticism is defined as a tendency toward negative self-esteem that leads to feelings of worthlessness, failure, and guilt when expectations are not met (Luoma and Chwyl, 2022).

Self-criticism may be associated with negative emotions, especially contempt and self-loathing. Research shows that this strength of negative emotions toward oneself and the inability to adequately cope with them puts overly self-critical people at risk for developing and maintaining depressive states (Ehret et al., 2015; Iancu et al., 2015; Schoon and Mortimer, 2017). In previous studies, self-criticism in adolescents has been analyzed in some clinical samples with more severe depressive symptoms, proving a direct link between the two processes (Castilho et al., 2015; Löw et al., 2020). When young people are emotionally distressed in difficult situations, or are not performing well, they may be self-compassionate or self-critical. They often engage in self-critical egocentrism and rumination when they judge themselves harshly, think their difficulties are unique, and focus on their negative thoughts and feelings in ways that reinforce them (López et al., 2015). These processes are relevant to the emotional functioning of youth.

Self-criticism typically occurs when people notice failures in important life situations or in difficult situations and includes automatic harsh self-blaming and self-attack with outright anger, disgust, or even hatred (Peter and Gazelle, 2017). Self-criticism can take many forms and functions, which can focus on feeling inadequate, defeated, or focus on feeling disgusted and angry at oneself. This latter form of self-criticism seems to be more problematic among youth because it can be used as an attempt to eliminate, exclude, and persecute oneself (Xavier et al., 2016).

#### 1.1.2. A digital game-based learning

It is crucial to clarify the distinction between game-based learning and gamification in education to provide a clear foundation for the research study. Digital game-based learning refers to an educational approach that is based on the use of video games and interactive digital simulations as the main learning process (Behnamnia et al., 2022). In digital game-based learning, the game itself is designed to provide educational content and learning objectives. It involves the creation of specially designed educational games that meet specific learning outcomes (Cai et al., 2022). The main goal of digital game-based learning is to promote active and engaging learning where learners learn through gameplay, problem solving and decision making in the context of the game.

On the other hand, gamification in education involves applying game elements, mechanics, and principles to non-game contexts to increase engagement and motivation in learning activities (Manzano-León et al., 2021). Rather than creating complete educational games, gamification integrates game features into existing educational materials or processes. Gamification in education offers many benefits such as more fun, a more relaxed atmosphere, marked progress in performance, and increased levels of accountability (Swacha, 2021). Because learning occurs through intermediate attitudes or behaviors, games must be matched depending on the context. For example, the use of more specific rules or goals in games can increase motivation to learn (attitude), while the learner’s cognitive strategies (behavior) will be enhanced by tailoring the game to learners’ abilities (Ofosu-Ampong, 2020). Gamification can be applied to the learning process effectively and engagingly. For example, Kahoot! has been evaluated as an approach to learning that is useful and enjoyable for students (Yanuarto et al., 2023). It can be used as an instructional strategy that incorporates gamification elements to enhance student achievement. Using Kahoot! for educational purposes has become a popular and effective way to engage students in active learning and improve their understanding of various subjects (Rajabpour, 2021). Kahoot! - is a game-based interactive learning platform that allows teachers to create quizzes, discussions, and surveys to assess and reinforce students’ knowledge. When using Kahoot! for educational purposes, instructors should consider specific learning objectives, age groups, and subject matter to develop effective and engaging quizzes (Resmayani and Putra, 2020). In addition, it is important to strike a balance between fun and learning so that Kahoot! activities remain educationally relevant while fostering student enthusiasm for the subject matter.

Since young people have a wide range of knowledge and social experience, they can master a set of integrated knowledge and skills that can be transformed into the concept of “competence.” In this case, teachers can outline the tasks of forming complex mental competencies based on logical, problem-critical thinking (Maratou et al., 2016). At present, digital technologies have gradually become an integral part of life. Digital learning, with its distinctive nature of being free from the constraints of time and space, increases the motivation to learn more than regular lectures. It also facilitates the ability to solve problems and thus ensures optimal learning outcomes (Sung et al., 2017). In addition, in the context of studying classical Chinese literature, it has been proven that students retain stronger learning motivation and develop not only better deep learning strategies and concepts, but a greater acceptance of technology when used with game-based learning than with the traditional approach in which tutorials such as power point slides and video clips (Yeo et al., 2022). Digital games are the main form of entertainment, and children and teenagers are spending more and more time playing them.

The development of self-critical abilities and values of students using digital games-based learning is an important and evolving area of research in education. Digital games have the potential to engage students actively, providing immersive and interactive learning experiences (Hashim et al., 2019). One such study examined how the integration of digital games in the classroom affected the critical thinking abilities of middle school students (Hussein et al., 2019). The critical thinking skills of students who participated in gaming activities were compared to those who followed traditional teaching methods. The results of the experiment showed that the computer game Ecoship Endeavour significantly improved students’ critical thinking skills. Another study examined students’ emotional experiences while playing games and their potential impact on decision-making processes (Denisova et al., 2020). The study sought to understand how emotions experienced in digital games contributed to the development of values and ethical considerations.

As games become more complex and open-ended, they seem to offer a formative experience that allows players to express themselves while developing their personality and knowledge. Digital technologies have a significant impact on users, prompting them to do new perceptual and cognitive activities. Given their audience and popularity, it’s natural to consider them an interesting alternative for delivering educational content (Cam and Tran, 2017). Students are introduced to the virtual world through games, and how they interact with technology can change the way they learn and the quality of knowledge they gain. The main reason for the success of the game is the toolkit that aids learning, as games provide students with a competitive platform to fully immerse themselves in the game (Mekler et al., 2016). It means that many educators are now choosing to use more stimulating ways of teaching.

### 1.2. Problem statement

This article is devoted to determining among Chinese youth the potential components of value orientations and goals in life, as well as the tendency to self-criticism with the use of digital games.

The study is motivated by the need to update data on contemporary values of young people with evidence base in the form of correlated statistical results. There is also a need to identify students’ propensity for depression due to high levels of self-critical skills.

The purpose of this study was to identify and describe the values of contemporary Chinese youth in the context of their future life goals and to analyze how self-criticism shapes students’ depressive tendencies with the use of digital game. Achieving this goal requires solving the following tasks: (1) collect and analyze data that students can provide about themselves with the help of the game Kahoot!; (2) identify among them the main life values and goals, to describe the dominant ones, taking into account the correlation of gender differences; (3) based on these responses, identify youths’ propensity for depression due to excessive self-criticism.

## 2. Methods and materials

### 2.1. Sample

The study was conducted on the basis of Shaanxi Technical College of Finance and Economics. The sample consisted of 157 Chinese students aged 18 to 25 years old. They were randomly selected among students of all courses and all majors. Their gender ratio was as follows: 109 girls and 48 boys.

### 2.2. Research design

**Figure 1:**
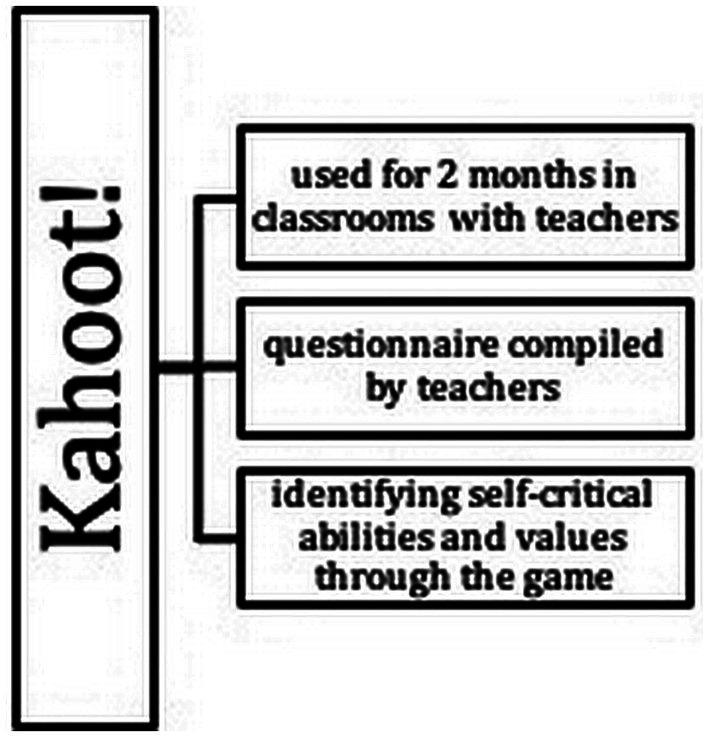
The course model.

The study was conducted in a Chinese cultural context using the method of experiment with a survey (Lenton, 2015), statistical (Goss-Sampson, 2019), and correlational (Clark and Watson, 2016; Mõttus and Rozgonjuk, 2021) data analysis. These methods provided the basis for qualitative and quantitative results. The students were offered a course that lasted 2 months. His task was to complete tasks with the help of games. The game chosen was Kahoot! It is a gamified platform that includes quizzes, discussions, and polls; a gaming platform for students to enable them to learn in a fun and competitive way instead of the traditional. Kahoot! encourages users to learn, collaborate and communicate in virtual classrooms, thereby creating a rich and active learning environment. The teachers created special questionnaires for this game. During the lesson, they demonstrated questions on the screen, and students had to answer questions using additional devices (tablet, smartphone or computer). Their goal was to determine the core values of their students. The course model is shown in Figure 1.

At the first study stage, students were questioned about the main life values, which they define for themselves. They were offered a number of questions with one answer choice. In total, there were 5 such practices for the entire experiment (including primary and repeated practices). The significance and relevance of these questions lie in their ability to capture significant aspects of respondents’ values, life goals, and emotions. They provide valuable information about people’s attitudes and inner world. Three questions are sufficient to achieve this goal.

Questions for the first test:

1. What is most valuable to you in life?
2. What life goals do you consider most important to you?
3. What feelings do you experience most often?

The validity of its content was verified by a discussion with university professors included in the study. The discussion took place via email. The final form of the questions was established after reaching a consensus among all participants regarding the completeness of the coverage of the issues under study, the problems, and the attitude of the respondents to them. The promptness of the survey was tested using Cronbach’s alpha method. The obtained result α = 0.769 allows us to assess the internal consistency of the survey as sufficient for the study.

### 2.3. Statistical processing

Then a correlation analysis of the obtained data was carried out, which allowed obtaining quantitative results about the level of self-criticism and self-esteem of the participants. Given the statistical data on life goals and values, the second stage consisted in determining the internal qualities of the students’ character. For this purpose, two models of comprehensive review of N/NE personality models (Mõttus and Rozgonjuk, 2021) and E/PE (Clark and Watson, 2016) were used. Focusing on the shape of N/NE model, the correlation between participants’ self-criticism and depressive state was determined, as they are directly related to each other. This model highlights a facet of individual differences in anxiety and fear. Although anxiety and fear are different in many similar models, they are often combined when assessing self-criticism. The second aspect is the aspect of depression, stress response, and vulnerability. The last component has a secondary load in the domain of hostility, anger, and aggression. Thus, these four components-anxiety, sadness, hostility, and vulnerability to stress-form the central facets of the N/NE used in the study. The integrative E/PE model synthesizes the core elements included in the current conceptualizations of the construct. This integrative model has four central aspects: belongingness (consisting of friendliness and sociability), positive affectivity or emotionality (joy and enthusiasm), energy and activity. In this model, two other aspects are also considered part of E/PE but play a less important role: entrepreneurialism and desire for change, as well as ambition. Empirical testing of the two models described above was conducted in an independent sample of university students.

There was also a statistical analysis of gender differences, which influenced the results.

### 2.4. Ethical issues

All processes performed in the study involving human subjects complied with the study’s ethical standards. Informed consent was obtained from all participants included in the study. Ethical issues were not violated during the study.

## 3. Results

The first study stage proved that students’ internal aspirations were much more important than external ones. On average, young people assessed health as a relatively important life value, while the pursuit of personal growth, position in society, and relationships were considered less important. All of these values were, on average, significantly more important than external aspirations for wealth, fame, or public image. Among the values, transcendent values (benevolence, reliability, decency, and caring) were the most important in the sample. Values of self-improvement, in general, were the least important. From this follows a low level of self-critical abilities. Willingness to change and self-preservation values were on a par with sincerity. Reflecting the traditional, collective values of the Chinese, linking moral behavioral characteristics and loyalty, the individualization of personal values receded into the background.

The survey data show that health has the greatest value for students. This was noted by 35% of those surveyed. After this come wealth and earnings (26%), then family (24%), education (5%), and friends (8%). Only 2% of respondents would like to start their own business in the future.

The majority of respondents believe that the main goal in life is to be healthy, this was noted by 37% of respondents. In second place is the family, so answered 20% of respondents. In third place is the desire to have a good education (12%). 12% consider it important to have good friends. 10% strive to earn a lot of money, and 9% want to build a successful career.

The questionnaire made it possible to reveal the level of self-critical abilities of students. Thus, 35% of respondents noted that their mood depends on the specific situation in life, 29% usually experience emotional lift and a sense of liveliness. Alarming is the fact that 7% experience a state of indifference and apathy, and 4% experience a state of unbalance and anxiety. The last two indicators are alarming, although this is a small number among the team. However, these students need help so that their depressive moods and self-criticism do not affect the learning process and mental and psychological health.

A post-test on the same question (3) gave the following results, which are presented in Table 1.

**Table 1:** Changing the level of self-criticism.

Self-critical abilities and feelings	Hard to answer	Joy	Stillness	Indifference and apathy	Anxiety
Pre-test	35%	29%	25%	7%	4%
Post-test	30%	38%	28%	3%	1%

Thus, an improvement in anxiety and apathy can be noted. They are minor, but using the Kahoot game as a regular testing tool has helped direct some students to work on themselves. It helped to trace the dynamics of their self-critical abilities. After completing the survey, it is convenient to discuss the findings with students individually. Determine who needs the help of a psychologist if the indicators of self-criticism are too low (tendency to depression, etc.). The game method has the advantage that students do not think about the seriousness of the questions and perceive them as playing through the game to get more points. It is not only a good tool for teaching but also the work of psychologists.

The results showed that digital games cannot influence the development of self-critical abilities of students, however, they can be a high-quality tool in psychological work with students to determine depressive moods, an overestimated level of self-criticism, and other problems that prevent them from learning. Based on these data, correlation analysis and analysis of average differences showed that the predominance of internal life aspirations among young people was associated with transcendent values, while their external aspirations were associated with the values of self-improvement. They arise against a background of self-criticism and dissatisfaction with certain aspects of one’s life. Internal desires and aspirations to develop were supported by moral progressivism. The results are presented in Table 2.

**Table 2:** Results of correlation analysis.

Scales	*N*	*n*	*k*
Internal values	0.63	0.36	0.2
External values	0.72	0.5	0.2
Relationships	0.78	0.51	0.5
Society	0.86	0.63	0.6
Personal growth	0.71	0.65	0.06
Health	0.75	0.59	0.4
Affluence	0.97	0.83	0.14
Self-improvement	0.67	0.45	0.9
Self-determination	0.33	0.21	0.12

The results of the second study phase showed that self-criticism, which is defined as a propensity for negative self-evaluation when expectations are not met, inherently influences the development of depression. The correlations between self-criticism and N/NE are strong (rs ¼ 0.50–0.65) and have a particularly strong association with the depressive aspect of youth. Self-criticism also has a weaker but moderate negative correlation with extraversion, from 0.30 to 0.40 (see Table 3). It was most correlated with the positive emotional component of E/PE. Perfectionism is the basis for self-criticism in young people. It is characterized by the formation of excessively high standards of performance. In addition, many definitions of perfectionism include a tendency to have negative emotional reactions that follow the failure to meet one’s standards. This tendency shows a close empirical association with self-critical abilities. The strongest correlate of maladaptive perfectionism is N/NE, with correlations ranging from approximately 0.30 to 0.60, depending on the measure. Specific aspects of N/NE inherent in the study participants are anxiety and anger, hostility, depression, vulnerability, and self-consciousness. Some evidence of an inverse relationship with E/PE was found (especially positive emotionality, benevolence, and conscientiousness). Adaptive perfectionism is most strongly correlated with conscientiousness and has moderate correlations with all facets of N/NE and E/PE. Hard work has a strong correlation with adaptive perfectionism. Thus, perfectionism clearly has an important place in the personality hierarchy of young people. Non-adaptive perfectionism is very similar to self-criticism, characterized primarily by high levels of N/NE (sadness and depression) and low E/PE. Adaptive perfectionism is associated with high levels of student conscientiousness, especially in the context of industriousness, high levels of energy, and assertiveness of E/PE components. Given the sample size, all of the character facets that are responsible for the development of self-criticism had significant positive correlations with each other (*p* < 0.001). Using common metrics for correlation size, 86% of correlations between strengths were average (≥0.30) and 24% were large (≥0.50), indicating significant overlap between strengths.

**Table 3:** Analysis for gender effect on results.

Scales	Women	Men	*p*
Internal values	0.61 (0.63)	0.28 (0.36)	0.0001
External values	−0.73 (0.72)	−0.35 (0.5)	0.0003
Relationships	0.55 (0.78)	0.25 (0.51)	0.0057
Society	0.59 (0.86)	0.22 (0.63)	0.0033
Personal growth	0.55 (0.71)	0.30 (0.65)	0.0386
Health	0.73 (0.75)	0.34 (0.59)	0.0008
Affluence	−0.49 (0.97)	0 (0.83)	0.0021
Fame	−0.62 (0.98)	−0.32 (0.65)	0.0246
Self-improvement	−0.67 (0.63)	−0.45 (0.54)	0.0282
Self-determination	0.33 (0.29)	0.21 (0.24)	0.0086

Because of the possibility that gender could influence the indicators, a correlation analysis of gender with the potential values of youth identified in the first step of the questionnaire was conducted. The data are presented in Table 3. For girls, intrinsic values were more important than extrinsic values. For girls, health and social contribution were important, while for boys - prosperity. Self-improvement was higher on average for boys, but self-overcoming values were higher for girls.

Based on the statistical and correlational data obtained, one can conclude that Chinese students of 18–25 years old most strongly value and strive to build strong relationships with family and friends and to have good health. Young people who sought self-development did not share any special values other than career and earning money. One could note a fairly progressive assessment of contemporary youth values and goals. Much of this progressivism is defined on the basis of the pursuit of personal growth, which was consistent with concern for others. The only other significant correlation was negative between wanting to contribute to society, but following and obeying authority figures and cultural traditions. The participants who showed an active desire for development were focused on their own psychological needs. This led them to be overly perfectionistic and self-critical. These qualities can often lead young people to a depressive state. At the end of the study, none of the participants were identified as having this condition during the interviews and analyses. Negative emotions and moods were noted, which may indicate the possible development of depression due to dissatisfaction with oneself and one’s life. Teachers, friends, and parents should be more attentive and lenient to such students and ask for help in case of deterioration.

## 4. Discussion

Speaking of self-criticism, in a 6-month study involving 64 children (ages 8 to 16), they were categorized as vulnerable in the interpersonal sphere. They were more likely to have increased depressive symptoms after domain-congruent rather than domain-incongruent stressors (Auerbach, 2015). A three-month prospective study of 486 high school students also found that feeling needed, one component of social therapy, predicted increased depressive symptoms in girls and boys regardless of the stressor. In a 12-month prospective study of 129 adolescents, there was a need for and connection with negative peers, but not in academia (Auerbach et al., 2014). In this case, neither achievement orientation nor self-criticism predicted changes in depression.

Another study assessed differences in character strengths among adolescents ages 10 to 17 using the VIA-Youth questionnaire. Results showed that most character strengths were associated with significant trends across age groups (Soto and Tackett, 2015). Many of the differences in character strengths reflected a decline as they matured, consistent with the personality developmental disorder hypothesis and empirically demonstrated by the decline observed in previous work. Conversely, consistent with the principle of adulthood (Roberts and Nickel, 2017), three character strengths showed higher levels at older ages (beauty, modesty, and perspective). All three of these strengths are quite personal, perhaps suggesting a strengthening of the inner life of youth (Brown et al., 2020). In a study conducted with Chinese students, personality traits and character traits also had a significant effect on the results. They helped to identify the students’ core values, among which health was at the top of the list for both boys and girls. Girls in particular tend to become more self-critical during adolescence, which is consistent with the present study findings of lower levels of character strengths. This finding could potentially reflect several processes, including increased self-awareness during adolescence. Although both boys and girls become more self-aware during adolescence, the earlier onset of puberty in girls may lead them to be more self-aware early in life, which may manifest in insecurity compared to boys. This difference may reflect differences in response styles. Boys’ responses could serve to appear attractive or socially desirable to girls.

In the context of character strengths, adolescents may perceive themselves as lacking sufficient social resources to recognize themselves as capable of having character strengths. These findings are consistent with both disorder and the emergence of personality maturation in early adulthood. Although adolescents are likely to go through a period of maturation, these changes may nonetheless be slow, as other personality traits undergo only a 10 percent change in late adulthood. Another study focused on entrepreneurial skills in adolescents to determine the relationship between the dependent variable and key factors in their development: personal, social, and educational (independent variables; Campo-Ternera et al., 2022). The results showed that the great influence of potential entrepreneurial ability on effective entrepreneurial activity is determined by the direct influence of personal qualities and life skills, as well as family.

Another study examined the impact of digital role-play learning on the critical thinking ability of high school students (Chen and Wu, 2023). A digital game was developed whose storylines presented issues of critical thinking. The results of the study showed that the student participants made significant progress in both overall critical thinking performance and learning motivation. Similar studies on the effectiveness of digital game learning among students were also compared instead of the results of the present study.

Another study examined cooperative learning and self-criticism skills among students who used their mobile phones as a means to play a digital game (Lee et al., 2016). The results showed that group plays in VR helped increase motivation to learn and develop healthy self-criticism skills. The study attempted to contribute to relevant discussions with an innovative approach in which digital games were seen as a valid way for users to apply a variety of analytical skills and critical thinking to the gameplay and therefore improve critical thinking. For example, users had to provide the maximum advantage to win the game by using all their critical thinking abilities, such as guess identification, logical interpretation, and argument evaluation, whenever necessary.

### 4.1. Limitations

However, there are a number of limitations to this study. It could not determine the influence of the participant’s upbringing, family environment, and personal goals, which may have been hidden, on the study results. It follows that the statistics obtained may be altered due to individual student reasons, which were difficult to ascertain with the questionnaire.

## 5. Conclusion

The study examines multiple aspects of the research findings, including internal and external aspirations, values, self-critical abilities, tendency to depression, and perfectionism. Each phase of the study is discussed separately, allowing for a comprehensive examination of the results. The results are also contextualized by discussing the significance of different values in the cultural and social context of Chinese students. The conclusions are in line with the objectives and show that for 35% of the participants the main life value is health, for 26% - earnings, and the formation of happy family relationships is a priority for 24%. These data are confirmed by the results showing the main goals of the participants. These also include their health (37%), the desire to have a family (20%), education and friends (12% each). For girls, internal values were more important than external ones. Health and social contribution were mostly important for girls, and wealth - for guys. Alarming is the fact that 7% experience a state of indifference and apathy, and 4% experience a state of unbalance and anxiety. This indicates a possible tendency for students to be depressed due to an increased level of self-criticism. Self-criticism also had a weaker but moderate negative correlation with extraversion of 0.30 to 0.40. It turned out to be most correlated with the positive emotional component of E/PE. Thus, no clear and serious reasons for the development of depression in young people were revealed in the analysis. These results have implications for further research in education, as it may provide a basis for developing and improving new methods of constructing curricula.

## Data availability statement

The original contributions presented in the study are included in the article/supplementary material, further inquiries can be directed to the corresponding author.

## Ethics statement

The studies involving humans were approved by Shaanxi Technical College of Finance and Economics. The studies were conducted in accordance with the local legislation and institutional requirements. The participants provided their written informed consent to participate in this study.

## Author contributions

The author confirms being the sole contributor of this work and has approved it for publication.
